# Abnormal Connectivity Within Anterior Cortical Midline Structures in Bipolar Disorder: Evidence From Integrated MRI and Functional MRI

**DOI:** 10.3389/fpsyt.2019.00788

**Published:** 2019-10-29

**Authors:** Jie Yang, Weidan Pu, Xuan Ouyang, Haojuan Tao, Xudong Chen, Xiaojun Huang, Zhening Liu

**Affiliations:** ^1^Institute of Mental Health, The Second Xiangya Hospital, Central South University, Changsha, China; ^2^Medical Psychological Center, The Second Xiangya Hospital, Central South University, Changsha, China; ^3^Medical Psychological Institute, Central South University, Changsha, China

**Keywords:** bipolar disorder, functional connectivity, structural connectivity, machine learning, multimodal fusion

## Abstract

**Background:** Aberrant functional and structural connectivity across multiple brain networks have been reported in bipolar disorder (BD). However, most previous studies consider the functional and structural alterations in isolation regardless of their possible integrative relationship. The present study aimed to identify the brain connectivity alterations in BD by capturing the latent nexus in multimodal neuroimaging data.

**Methods:** Structural and resting-state images were acquired from 83 patients with BD and 94 healthy controls (HCs). Combined with univariate methods conducted to detect the dysconnectivity in BD, we also employed a semi-multimodal fusion framework fully utilizing the interrelationship between the two modalities to distinguish patients from HCs. Moreover, one-way analysis of variance was adopted to explore whether the detected dysconnectivity has differences across stages of patients with BD.

**Results:** The semi-multimodal fusion framework distinguished patients from HCs with 81.47% accuracy, 85.42% specificity, and 74.75% sensitivity. The connection between the anterior cingulate cortex (ACC) and superior medial prefrontal cortex (sMPFC) contributed the most to BD diagnosis. Consistently, the univariate method also identified that this ACC–sMPFC functional connection significantly decreased in BD patients compared to HCs, and the significant order of the dysconnectivity is: depressive episode < HCs and remission episode < HCs.

**Conclusions:** Our findings, by adopting univariate and multivariate analysis methods, shed light on the decoupling within the anterior midline brain in the pathophysiology of BD, and this decoupling may serve as a trait marker for this disease.

## Introduction

Bipolar disorder (BD) is a debilitating and common mental disease, affecting approximately 2% of the global population (1). Patients often struggle with significant functional impairments including unemployment, high health-care utilization, and social stigma (2). Neuroimaging studies of BD have reported reduction of gray/white matter density in distributed areas throughout the brain (3, 4), possibly reflecting that BD involves alterations in structural brain networks. The alterations in widespread structural brain networks are paralleled with disrupted functional brain networks in BD, evident across multiple neuronal systems involved in emotion regulation, high-order cognition, and self-referential functions (5–7).

A long-standing hypothesis is that functional links are underlined by anatomical connections in the brain, which has been supported by recent evidence showing that the network structure of the cerebral cortex shapes the functional connectivity on multiple time scales (8). Prior studies have highlighted that the structural and functional connectivity abnormalities are interrelated in BD (9) after observing the association between the decreases of functional connectivity and impaired white matter integrity in the cingulate–amygdala circuit (10). An emerging notion suggests that the structural brain network can be manifested by the white matter axons between brain areas and the morphometric correlations within gray matter (GM) regions (11, 12). Morphometric correlations likely reflect anatomical connectivity, which has been evidenced by a study highlighting the apparent system-specific correlation patterns between cortical GM and underlying white matter connectivity (13). Furthermore, morphometric correlations are closely related to the white matter fibers and coupling of functional activity between corresponding brain areas. Thus, combining the morphometric network and functional network as an innovatively multimodal approach may help to further illuminate the neuropathology of BD.

Another multivariate analysis method attracting extensive attention is the application of machine learning in BD study, which can utilize the neuroimaging data as features to individually distinguish patients with BD from healthy controls (HCs) and therefore be able to translate the clinically relevant structural and functional brain connectivity into objective and clinically useful biomarkers (14, 15). For example, by using the functional pattern of the inferior frontal gyrus, Roberts et al. discriminated adolescents with BD and individuals at genetic high risk from HCs, with 64.8% average accuracy (14). Moreover, Mwangi et al. utilized the GM density and white matter density to differentiate patients with BD from HCs and reported a prediction accuracy of 70.3% and 64.9%, respectively (15). Although these studies have made a significant progression in establishing the model of building bridges between aberrant brain function/structure and clinical expression in BD, several limitations should be taken into account for future studies. First, these studies conducted the classification based on restricted regions of interest, leading to the inability to detect abnormal functional architecture of BD at the whole-brain level. Moreover, these studies considered the brain structure and function in isolation and ignored fusing the complementary information from each other, which is regarded as a possible source leading to a relatively low classification accuracy rate under 80%, the criterion considered clinically useful (16).

Therefore, for the current study, we employed a semi-multimodal fusion approach that was proposed in our prior study to find the discriminative brain connectivity pattern in BD (17). The approach exploits the interactive information of the structural MRI and resting-state functional MRI (fMRI) data by comprehensively considering the abnormal connectivity pattern due to their associations of disease from both the structure and function aspects. In this approach, whole-brain structural connectivity was constructed by the correlations between the GM volumes within each brain area across subjects, and whole-brain functional connectivity was constructed by the correlations between the mean time courses of each pair of region of interest (ROI). It utilized the distance of brain functional and structural connections as one of the feature selection criteria in this multivariate analysis method. As it comprehensively considers the abnormal brain connectivity pattern due to their associations of disease from both structural and functional aspects and the inter-modality latent nexus, our approach may provide great potential to increase the classification accuracy and enhance the credibility of the detected biomarkers.In parallel with the semi-multimodal fusion approach, we also used the traditional univariate statistics method to investigate whether the results obtained from the framework would yield consistency in the between-group comparison.

## Materials and Methods

All study procedures were approved by the medical ethics committee of the second Xiangya Hospital, Central South University. Prior to obtaining consent, the capacity to provide informed consent for all potential participants was ascertained by two licensed psychiatrists. After explaining the study procedures, informed written consent was obtained from all participants. All study procedures were conducted in strict accordance with the Declaration of Helsinki.

### Study Sample

In this study, 92 patients with BD and 98 demographically similar HCs were recruited by the Second Xiangya Hospital, Central South University. All participants were right-handed native Chinese speakers who were carefully screened in a semi-structured interview by two trained senior psychiatrists. Patients were confirmed to meet the *DSM-IV* (*Diagnostic and Statistical Manual of Mental Disorders*) criteria for bipolar I disorder or bipolar II disorder (18). Diagnostic procedures included patient history information gathered from patients and their families. The medical, neurological, and psychiatric examinations were carefully performed by clinical psychiatrists. Patients with BD were excluded if they met the following criteria: 1) less than 18 years old or greater than 45 years old; 2) previous electroconvulsive therapy and any other contraindications to MRI; 3) history of alcohol or substance abuse except nicotine; 4) chronic neurological disorders or debilitating physical illness; and 5) benzodiazepine treatment, if any, stopped less than 24 h prior to scanning.

HCs were physically healthy, had no history of severe illness, and had no first-degree relative with a history of psychiatric illness. Clinical assessments for the HCs were conducted by an experienced psychiatrist to verify inclusion and exclusion criteria. Eligible participants were required not to meet the *DSM-IV* criteria for an axis I psychiatric disorder, non-patient edition (18), and had to meet exclusion criteria (1), (2) and (3).

### Clinical Characteristics

Clinical symptoms were assessed with the 17-item Hamilton Depression Rating Scale (HAMD) (19), Hamilton Anxiety Rating Scale (HAMA) (20), and Young Mania Rating Scale (YMRS) (21) for patients with BD. Details are presented in **Table 1**. Patients whose HAMD score ≥ 17 and YMRS < 12 were defined as depressive episode, patients whose YMRS score ≥ 12 and HAMD < 17 were defined as mania or hypomania episode, patients whose YMRS score ≥ 12 and HAMD ≥ 17 were defined as mixed episode, and patients whose HAMD score < 17 and YMRS score < 12 were defined as remission episode.

**Table 1:** Demographic and clinical data.

Items	Patients With BD(N = 83)	Healthy Controls (N = 94)	T/χ2 Value	*P*-Value
Age (years)	25.63 ± 5.59	23.3 ± 4.6	3.1	0.002**
Gender (M/F)	37/46	47/47	0.52	0.47
Education (years)	13 ± 2.9	14 ± 2.1	-2.3	0.02*
HAMD	11.1 ± 9.3	N/A	N/A	N/A
HAMA	9.3 ± 8.7	N/A	N/A	N/A
YMRS	6.6 ± 9.6	N/A	N/A	N/A
Age of onset (years)	21.4 ± 5.3	N/A	N/A	N/A
Total duration (months)	53.7 ± 57.1	N/A	N/A	N/A
Current mood				
Manic	19	N/A	N/A	N/A
Depression	35	N/A	N/A	N/A
Remission	29	N/A	N/A	N/A
Mixed	0	N/A	N/A	N/A
Medication administration				
Drug naive	5	N/A	N/A	N/A
Mood stabilizers	3	N/A	N/A	N/A
Mood stabilizers + antipsychotics	50	N/A	N/A	N/A
Mood stabilizers + antidepressants	8	N/A	N/A	N/A
Mood stabilizers + antipsychotics + antidepressants	7	N/A	N/A	N/A
Antipsychotics + antidepressants	8	N/A	N/A	N/A

P-value was calculated by independent two-sample t-test. BD, bipolar disorder; N, number; HAMD, Hamilton Depression Rating Scale 17 item; HAMA, Hamilton Anxiety Rating Scale; YMRS, Young Manic Rating Scale; N/A, not available.

*p < 0.05 and **p < 0.01.

#### Data Acquisition

Imaging data were collected on a 3-T Philips Gyroscan Achieva scanner. High-resolution three-dimensional T1-weighted scans were recorded in a magnetization prepared rapid gradient echo sequence [Time of repetition (TR)/Time of echo (TE) = 1,924/20 ms; Field of view (FA) = 8°; acquisition matrix = 256×256; FOV = 250×250 mm^2^]. Whole-brain resting-state fMRI data were acquired using a gradient-recalled echo-planar imaging pulse sequence (TR/TE = 2,000/25 ms; FA = 90°; acquisition matrix = 64×64; FOV = 24×24 cm^2^; total volumes = 250). During the 8 min resting-state fMRI scan, participants were simply instructed to keep their eyes closed, relax, lay still in the scanner, and refrain from falling asleep.

### MRI Data Analysis

MRI data analyses were carried out using Statistical Parametric Mapping (SPM8: http://www.fil.ion.ucl.ac.uk/spm). The preprocessing flow is in accordance with the standard voxel-based morphometry–diffeomorphic anatomical registration through exponential lie algebra (VBM-DARTEL) procedure. First, T1 images were segmented into three tissue types (GM, white matter, and cerebrospinal fluid) using the standard unified segmentation module in SPM8. Second, study-specific GM templates were derived from the entire image data set using the DARTEL method. Third, after initial affine registration of the GM DARTEL templates to the corresponding tissue probability maps in the Montreal Neurological Institute (MNI) space, non-linear warping of GM images was performed to match the corresponding MNI space DARTEL GM templates. Fourth, images were modulated to ensure that relative volumes of GM were preserved following the spatial normalization procedure.

We constructed the structural connection matrix as the following steps. First, we generated 90 cortical and subcortical ROIs by applying the automated anatomical labeling (AAL) parcellation scheme (excluding the cerebellum). Pearson correlation coefficients (CCs) were calculated between the volumes within each ROI across subjects. Therefore, two structural connectivity networks (BD group vs. HC group) were generated based on the aforementioned approaches. After removing 90 diagonal elements, we extracted the lower triangle elements of the CC as features; the feature space was spanned by the (90×89)/2 = 4,005-dimension feature vectors. Therefore, 4,005-dimension feature vectors *M_S_^BD^* and *M_S_^HC^*were constructed for the BD and HC group, respectively.

### Resting-State fMRI Data Analysis

Resting-state fMRI data were analyzed using SPM8 software and the functional toolbox data processing and analysis for brain imaging (DPABI) (22). The preprocessing flow is in accordance with the standard processing procedure which was implemented in DPABI. Before functional image preprocessing, we discarded the first 10 volumes of each participant to allow the MR signal to reach equilibrium. The remaining 240 fMRI volumes were preprocessed as the following steps including slice-time, realignment, coregistration, normalization into the MNI space, resampling at 3 mm^3^, and spatial smoothing with full width at half maxima (FWHM) = 8 mm. We treated nuisance covariates including white matter signals and cerebrospinal fluid signals as confounding factors to regress out to minimize non-neural influences on fMRI signals. We controlled for head motion first through regression of six head motion parameters plus their temporal first derivatives. We used so-called scrubbing by removing outlier volumes, defined as frame-wise displacement (FD) of more than 0.5 mm from the previous frame or global mean intensity of more than 2 SDs. If more than 48 volumes were scrubbed (i.e., > 20% of the acquired volumes), we excluded these subjects from subsequent analysis. We also compared the motion parameters of these two groups, and we did not detect a significant difference of mean FD power value (patients with BD mean (SD) = 0.15 (0.12); HCs mean (SD) = 0.13 (0.07); p = 0.13). Finally, the residual time series was temporally band-pass-filtered (0.01–0.08 Hz) to reduce the effect of low- and high-frequency physiological noise (23). After excluding 9 patients with BD and 4 HCs because of their high motion parameters, there were 83 patients with BD (patients with BD type I = 79; patients with BD type II = 4) and 94 HCs enrolled in the subsequent analysis.

In the construction process of the functional connection matrix, we also applied the AAL parcellation scheme to parcellate all brain maps into 90 cortical and subcortical ROIs (excluding the cerebellum). Pearson correlations were calculated between the mean time courses of each pair of ROIs. We converted the resultant CCs to normally distributed scores by using the Fisher z transformation, and the variance due to the linear effects of age, gender, and education years was removed to derive the corrected symmetric matrix. Therefore, a 90×90 symmetric matrix was obtained for each participant. Finally, we extracted the lower triangle elements of CCs as features, and the feature space was spanned by the (90×89)/2 = 4,005-dimension feature vectors.

### Statistical Analysis

As the structural connections were defined by the Pearson CCs between the inter-regional volume across subjects within a group, we adopted a non-parametric permutation test with 10,000 repetitions to characterize the disease-associated structural networks of BD. Before conducting the non-parametric permutation test, a linear regression analysis was applied at every ROI for controlling the effects of age, gender, and education. The residuals of this regression were then substituted for the raw ROI volume values and described as corrected regional volumes. In each repetition, we randomly reassigned the corrected regional volumes of each subject to one of the two groups with the same number of subjects as were in the original groups and obtained an association matrix for each randomized group. Differences in CCs between randomized groups were then conducted to achieve a permutation distribution of difference under the null hypothesis. Lastly, the actual group-related difference of the CCs was assessed in the corresponding permutation distribution, and a two-tailed p-value was calculated on the basis of its percentile position.

A two-sample t-test was conducted to compare the corrected functional connectivity between the two groups (i.e., patients with BD vs. HC). Statistical maps of structural and functional connectivity were generated after multiple comparison analysis false discovery rate (FDR) corrected using the Benjamini and Hochberg method with *p* < 0.05).

### Machine Learning

**Figure 1:**
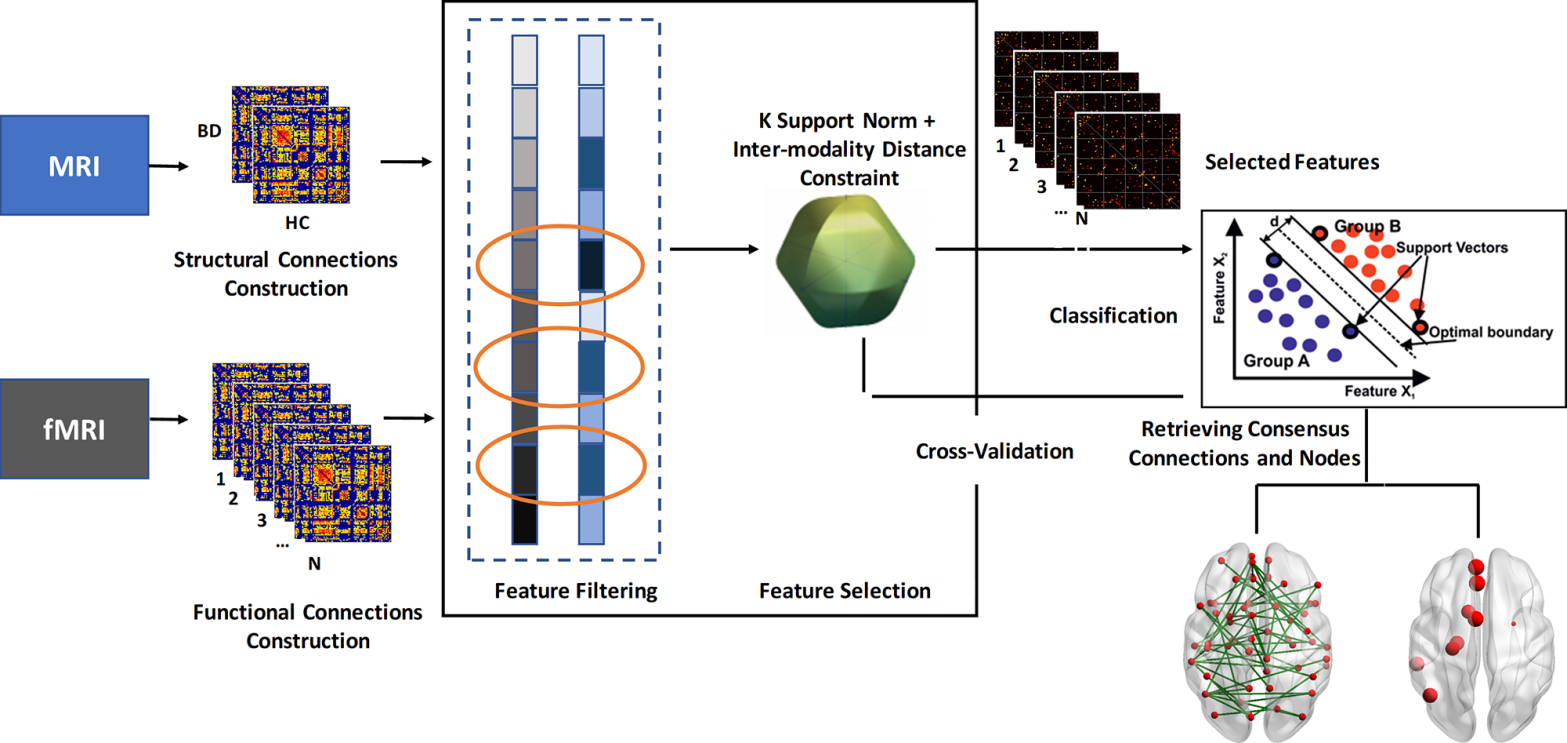
Flowchart of the multivariate analysis method in this study. BD, bipolar disorder; HC, healthy controls.

Except for the traditional univariate analysis, we also conducted a multivariate analysis method to further capture the latent and subtle discriminative brain connectivity pattern between patients with BD and HCs. The pipeline of the multivariate analysis method is presented in **Figure 1**. To avoid the curse of dimensionality of mass features that emerged in the neuroimaging field, a feature selection framework as suggested in a previous work, which is comprised of the initial feature filtering procedure and the modified sparsity regularization, was employed to remove redundant features before classification (17).

### Feature Filtering Procedure

The flow path of the feature filtering procedure was as follows:

1. The differential structural connection matrix Δ*M_S_*was generated by subtracting the corrected features in *M_S_^HC^* from the corresponding corrected features in *M_S_^BD^*, i.e., Δ*M_S_* = |*M_S_^BD^* − *M_S_^HC^*|.
2. All features in Δ*M_S_* were sorted in descending order in accordance with their absolute values.
3. All features of the corrected functional connection matrix were ranked according to their significance level in the two-sample t-test that was performed between patients with BD and HCs.
4. To integrate the corrected structural and functional connection information extracted from T1 and the resting state fMRI (rs-fMRI), we conducted an overlapping pattern to select the features. We selected the top-ranked c features from the different modalities, where c was set based on the rule that the value of the c-th feature in Δ*M_S_* equals the mean value of the whole Δ*M_S_* vector.
5. After overlapping the selected features from the two modalities, the final selected feature set L was obtained.

### Feature Selection Procedure

We assumed *X* = [*x*_1_…*x_i_ … x_n_*]*^T^* to be an *n*×1matrix that represents *l* features of *n* training samples, where the matrix *X* is constructed by the individual functional connectivity features extracted from the previous analysis. Let Y = [*y*_1_…*y_i_ … y_n_*]*^T^* be the *n* dimensional categorical target labels that we aim to predict (+1 = patients with BD; −1 = HCs). The linear regression model used for the prediction can be defined as follows:

$$\hat{Y}=\mathrm{XW}$$

where *W*∈*R^l^*^×1^ denotes the regression coefficient vector and $\hat{Y}$ indicates the predicted label vector. To fully utilize the complementary information conveyed by the latent nexus between structural and functional connectivity, an inter-modality distance constraint was added to the original k-support norm, a sparsity regularization that allows us to handle the curse of dimensionality for improving predictive performance. Additionally, compared with the commonly used least absolute shrinkage and selection operator (LASSO) and elastic net models, the k-support norm can give a better predictive performance, and it is useful for generating sparse but correlated features, which have been thought to be suitable for interpretation in medical research (24). Therefore, the object function of the adopted inter-modality feature selection framework can be further defined as follows:

$$\underset{w}{\text{min}}{\left\|\mathrm{XW}-Y\right\|}_{F}^{2}+\lambda_{1}{\left(\sum_{i=1}^{k-r-1}\left({\left|w\right|}_{i}^{↓}+\frac{1}{r+1}{\left(\sum_{i=k-r}^{d}{\left|w\right|}_{i}^{↓}\right)}^{2}\right)\right)}^{\frac{1}{2}}+\lambda_{2}D$$

where *λ*_1_ > 0 and *λ*_2_ > 0 control the sparseness and the degree of preservation of the inter-modality relationship, respectively, and *D* is the inter-modality distance constraint:

$$D=\sum_{i=1}^{n}{\left\|x_{i}-m_{i}\right\|}_{F}^{2}$$

where *x_i_* denotes the feature vectors of the functional connectivity features of the *i*-th subject and *m_i_*denotes the feature vectors of the anatomical connectivity features of the *i*-th subject. The relative distance between the inter-modality feature vectors is ${\left\|x_{i}-m_{i}\right\|}_{F}^{2}$. The detailed descriptions of the feature selection procedure are presented in the supplementary material (**S1**). Notably, both the feature filtering and feature selection procedures were only performed on the training data sets.

### Classification

We retrieved those features with non-zero regression coefficients for the subsequent classification using support vector machine (SVM). Here, we used the SVM of the radial basis kernel function, with parameter C = 10 to trade off learning and extendability, and other parameters were kept as default values (25). The general performance of the classifier was evaluated using a K-fold cross-validation procedure (K = 10). Specifically, we randomly divided the entire sample size into 10 sample sets; 1 set was first left out as a testing sample, and the remaining 9 sample sets were used for training the classifier. The optimal values of the regularization parameters including *λ*_1_ and *λ*_2_ with the best performances were determined based on the training data by searching on a mesh grid *G* (1:20:100) and were then used to classify the testing sample. Overall classification accuracy was assessed by calculating the proportion of the testing samples that were correctly predicted. To eliminate the randomness induced by the division step, we iterated the entire process 100 times and obtained the final classification accuracy by averaging the accuracies of the 100 rounds. The prediction accuracy, specificity, and sensitivity were calculated to evaluate the performance of the classifier.

### Consensus Connection and Node Definition

The absolute values of the regression coefficient vector *W* in the best trial and each round were summed to evaluate the discriminative ability of the corresponding connection to classification. Connections were defined as consensus connections when the corresponding regression coefficient *w* summarization value in all rounds ranked within the top 1% (4,005*1% = 40). Nodes were defined as consensus nodes when the corresponding regression coefficient w summarization value whose connections were listed in the consensus connection sets were arranged within the top 10% (90*10% = 9).

### Exploratory Analyses

Given that most of our patients were BD type I, we therefore conducted the univariate statistical analyses solely on the functional and structural connectivity of patients with BD I. The connections between the anterior cingulate cortex (ACC) and superior medial prefrontal cortex (sMPFC) selected by the semi-multimodal fusion framework were contributing most in distinguishing patients with BD from HCs. These findings were consistently supported by our univariate method analysis. As **Table 2** shows, all the strengths of the corrected functional connections between ACC and sMPFC of the two hemispheres (i.e., four functional connections that survived after FDR correction with p < 0.05) in patients with BD were reduced, compared with HCs. Considering the heterogeneity of our samples (that is, patients in the BD I group were currently at a different stage), we therefore allowed more granular groups in BD I and conducted statistical analysis. We divided patients with BD I into three groups, including depressive, mania, and remission episode, and assessed the actual group difference of the summation value of the four corrected functional connections by using one-way analysis of variance, followed by the Tukey–Kramer *post hoc* comparison procedure when significant main effects were present.

**Table 2:** After controlling for age, gender, and educational years, functional connections showing significantly altered connection strength in patients with BD compared to HC.

Connections	Patients with BD (N = 83)	Healthy controls (N = 94)	*P*-value	FDR corrected
**Corrected Structural Connections**				
None				
**Corrected Functional Connections**				
L ACC—L sMPFC	−0.1 ± 0.24	0.09 ± 0.23	<0.0001	0.0017
R ACC—L sMPFC	−0.09 ± 0.26	0.08 ± 0.26	<0.0001	0.0365
L ACC—R sMPFC	−0.09 ± 0.26	0.7 ± 0.24	<0.0001	0.0365
R ACC—R sMPFC	−0.1 ± 0.26	0.08+0.29	<0.0001	0.0494

FDR corrected using Benjamini and Hochberg method. L, left; R, right; ACC, anterior cingulate cortex; sMPFC, superior medial prefrontal cortex.

Therefore, in patients with BD, we consequently conducted partial correlation analysis between the summation value of the four ACC–sMPFC functional connections across two hemispheres with clinical variables (i.e., HAMD, HAMA, and YMRS) and used illness duration as a covariate.

## Results

### Demographic Characteristics and Clinical Symptoms

Demographic and clinical characteristics of patients with BD and HCs are presented in **Table 1**. There were no significant differences between the two groups with respect to gender. Patients with BD were significantly older than the HCs (*p* < 0.01), and the number of years of education received by patients with BD was shorter than that of the HCs (*p* < 0.01). Mean duration of illness was 53.7 months (SD = 57.1) for patients with BD.

### Statistical Analysis

**Figure 2:**
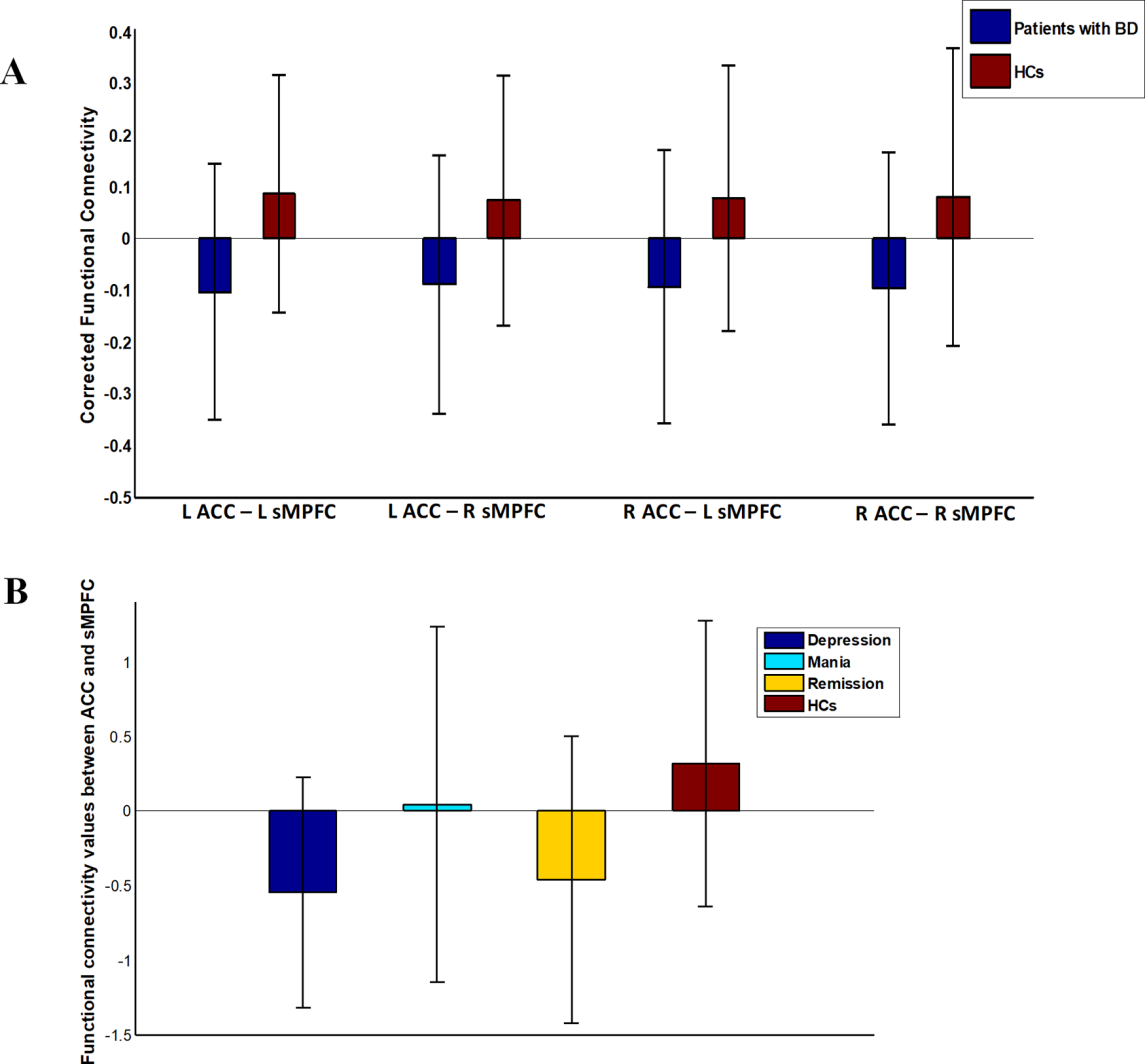
Functional connectivity in patients with bipolar disorder (BD) versus healthy controls (HCs). Error bars stand for ±1 standard deviation. **(A)** Patients with BD showed four significantly reduced functional connections between ACC and sMPFC (FDR corrected with p < 0.05); **(B)** There was an omnibus difference in the summation value of the aforementioned four abnormal functional connections across all diagnostic groups.

We did not detect any significant group-related differences in structural connectivity. As **Figure 2** and **Table 2** show, patients with BD displayed significantly altered functional connectivity between the ACC and the sMPFC when compared with the HCs. It should be noted that all the detected altered functional connections were reduced in patients with BD, compared with HCs.

### Consensus Connections and Consensus Nodes

**Figure 3:**
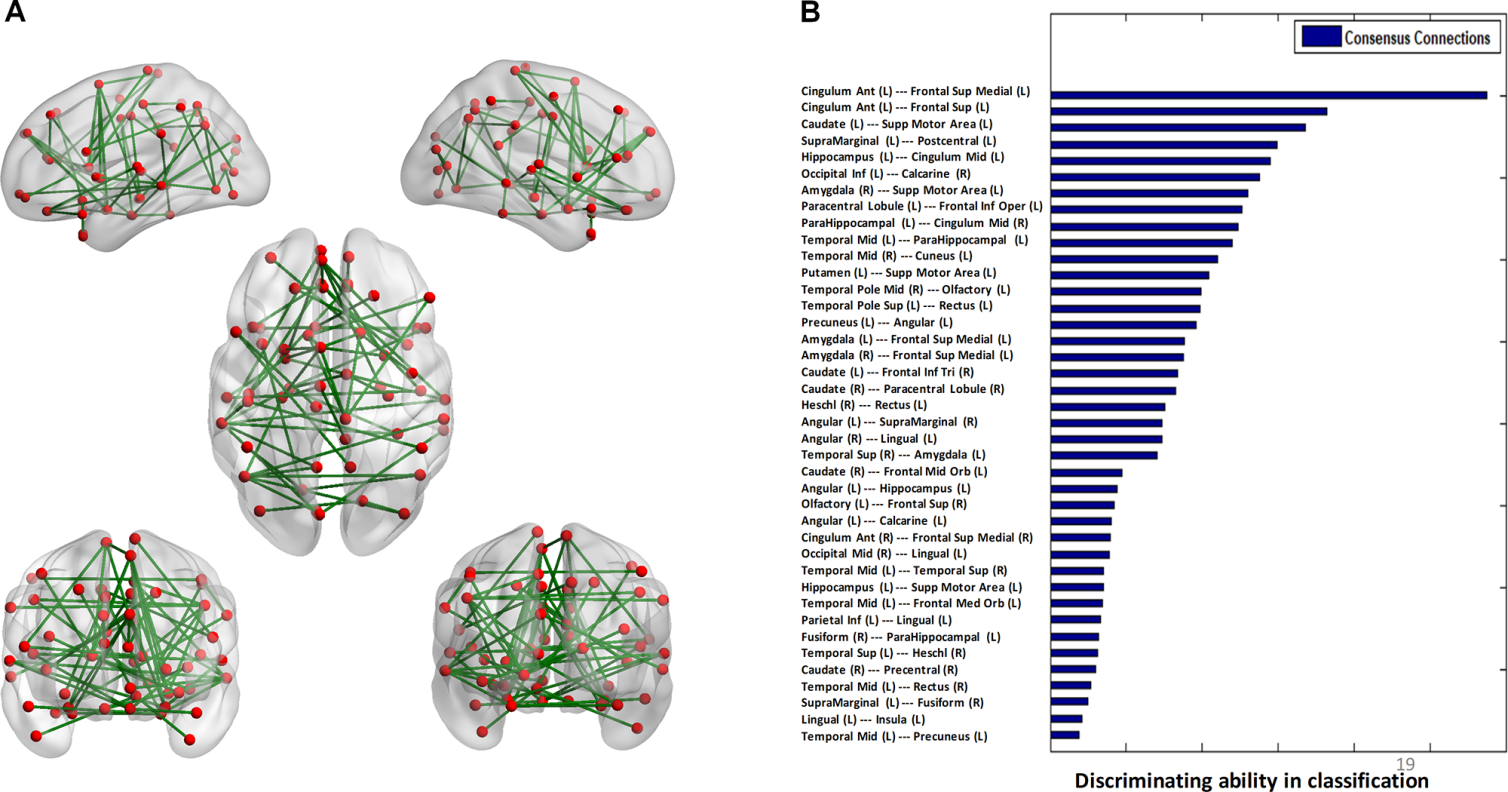
Consensus connections. **(A)** The spatial distribution of the consensus connections, whose weight was scaled according to their mean discriminative power in the tenfold cross-validation. **(B)** The normalized discriminative ability of the consensus connections.

**Figure 4:**
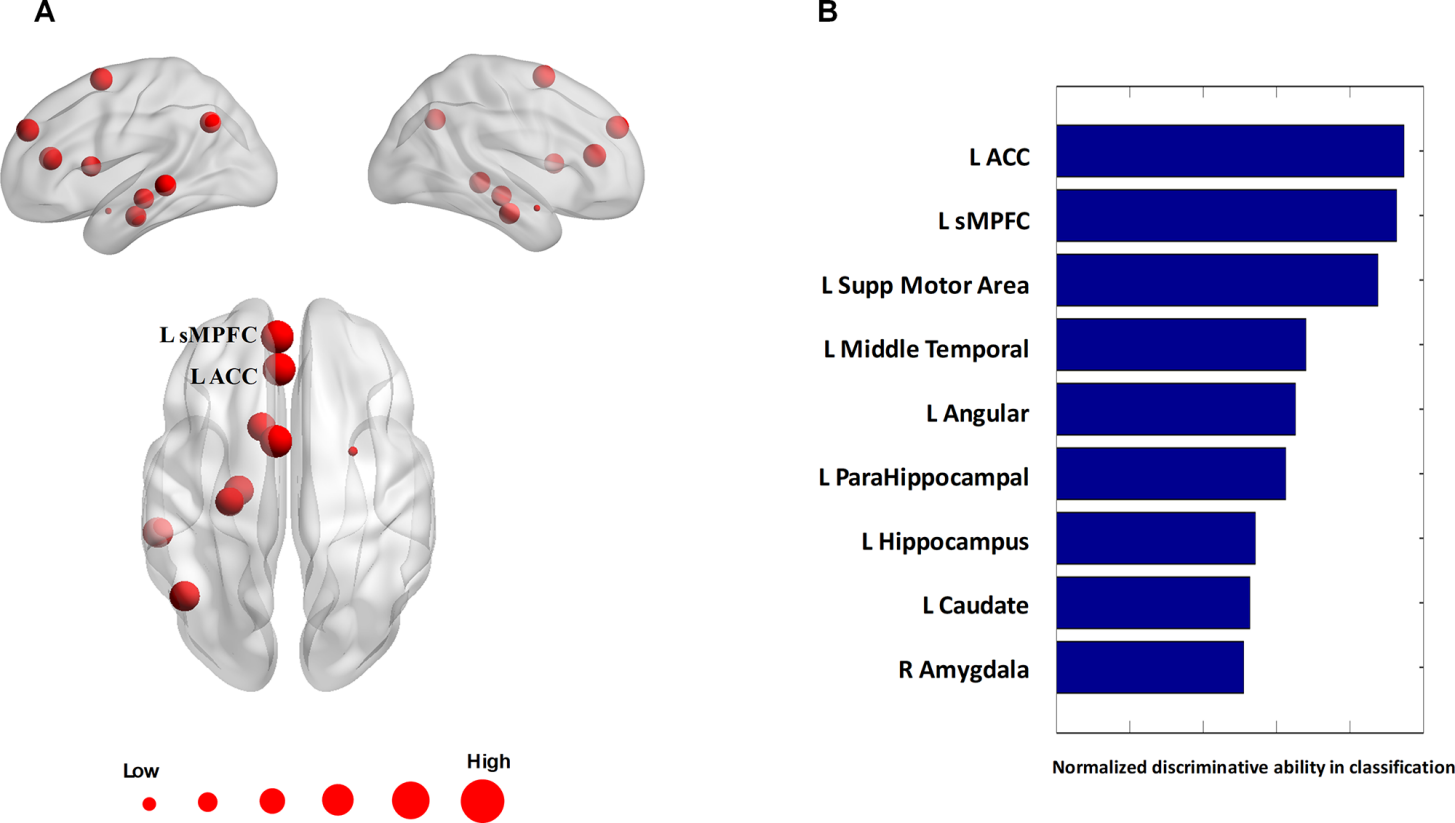
Consensus nodes. **(A)** The spatial distribution of the consensus nodes, whose size was scaled according to their mean discriminative power in the tenfold cross-validation. **(B)** The normalized discriminative ability of the consensus nodes.

The algorithm trained with whole-brain connectivity data distinguished patients with BD from HCs with accuracy = 81.47%, sensitivity = 74.75%, specificity = 85.42%, and area under the curve (AUC) = 0.88. The receiver operating characteristic curve is presented in **Figure S1**. Consensus connections and nodes were selected based on their best classification performance. The detailed spatial locations of the 40 selected consensus connections are presented in **Figure 3** and **Table S1**, and the corresponding information of the nine selected nodes is provided in **Figure 4** and **Table S2**. As **Figure 3** and **Table S1** present, the consensus connection set includes the connection between the ACC and the sMPFC, the connection between the ACC and the superior frontal cortex (sPFC), and connections between frontal regions (e.g., ACC and sMPFC) and the temporal and subcortical regions (e.g., middle temporal gyrus, amygdala, hippocampus, and caudate). It can be obviously observed that regions including the ACC, sMPFC, and supplementary motor area (SMA) were perceived to have the greatest discriminative ability in disease diagnosis.

### Exploratory Analyses

After re-analyzing the functional and structural connections solely in the patients with BD I, we observed that the results of the BD type I group were similar to those in whole sample (see **Table S3**). One-way analysis of variance revealed a significant omnibus difference in the summation value of the four corrected functional connections between ACC and sMPFC across all diagnostic groups [depressive episode mean (SD) = −0.55 (0.77), mania episode mean (SD) = 0.046 (1.2), remission mean (SD) = −0.46 (0.96), HCs mean (SD) = 0.32 (0.96), F = 9.32, *p* < 0.001; see **Figure 2**]. Post hoc tests revealed a significant decrease in depressive episodes compared with HCs (*p* < 0.001) and a decrease in remission episodes compared with HCs (*p* = 0.0011). We did not detect any significant correlation between the connectivity pattern of ACC and sMPFC with clinical variables in patients with BD.

## Discussion

The present study achieved good classification performance in discriminating patients with BD and HCs, with accuracy, sensitivity, specificity, and AUC of 81.47%, 74.75%, 85.42%, and 0.88, respectively. Besides the consensus connection between the ACC and sMPFC being found to contribute the most to BD diagnosis, the connection between the ACC and sPFC was also included in the consensus connection set. These findings were partly in accordance with the traditional univariate analysis, which showed the decreased functional connectivity between ACC and sMPFC in patients with BD compared with HCs. The ranking order of the detected ACC–sMPFC functional connectivity in all diagnostic groups is: depression episode < HCs and remission episode < HCs. Moreover, for selected consensus nodes, ACC, sMPFC, and SMA were identified as the most relevant brain regions for distinguishing patients with BD from HCs.

Notably, the semi-multimodal fusion framework employed in this study is a hierarchical design, which aims to elucidate robust and meaningful connections from a set of spurious connections. Specifically, the framework consists of feature filtering and feature selection procedures (17). In the feature filtering procedure, connections need to satisfy inter-group differences in both functional and structural aspects before they are picked for further analysis. In the feature selection procedure, the distance between the functional connection and its corresponding structural connection was used as the penalty term to constrain the overfitting in the machine learning algorithm. Therefore, by considering the inter-modality relationships between resting-state fMRI and structural MRI as feature selection rules, the selected biomarkers (i.e., consensus connections and nodes) in our study may be more reliable.

In this study, the most convergent finding from machine learning and traditional univariate analysis was located at the link between ACC and sMPFC, which both belong to the anterior midline of the brain. These findings of our study were consistent with previous studies showing decoupling within the midline of the anterior part of the brain in BD patients, compared with HCs (26, 27). The ACC has been involved in certain higher-level functions, such as attention allocation (28), reward anticipation (29), performance monitoring (30), and emotion regulation (31). Due to its anatomical, metabolic, and functional alterations during both the early stages and the different phases of BD (32–34), the ACC has been regarded as a trait marker of this disease. The sMPFC is critical for self-referential functions and regulation of emotion, behavioral, endocrine, and innate immunological responses to stress (35), whose abnormities in function and structure of sMPFC have also been highlighted to be associated with mood instability (36), the most challenging symptom in patients with BD.

Consistent with the aforementioned notion regarding the ACC functional alterations as a trait marker for BD, this study also found that the ACC–sMPFC decoupling was exhibited across depression and remission episodes in BD patients. We also observed the ACC–sMPFC decoupling in mania episodes, although this decoupling did not survive after the statistical correction. The absence of a significant difference may be due to our small samples with mania episodes, which limits the statistical power to detect a difference in this subgroup of patients. The ACC–sMPFC decoupling observed across different episodes of BD suggests that the altered functional connectivity of ACC with sMPFC, along with the ACC functional alterations, possibly serves as a potential trait marker for this disease. What should be noted is that since the majority of our patients are BD I subtype, it is strongly warranted for further study to examine the characteristics of this ACC–sMPFC coupling in patients with BD II, especially in those with mania episode.

Given some overlapped brain functions subserved by the ACC and sMPFC, such as self-referential functions, emotion regulation, as well as attention, how the coordination between the ACC and sMPFC affects emotion has attracted intense attention. After observing a series of results including a main effect of self-relevance in the MPFC and a main effect of valence in the ACC, Moran et al. concluded that emotional stimuli processed self-referentially at the MPFC are tagged for emotional valence *via* the ACC (37). Thus, we speculated that the frequent clinical observations in BD including the abnormal self-referential and emotional regulation may be associated with the decoupling of the ACC and the sMPFC, when the sMPFC over/under-generalizes self-relevant information or the ACC mislabels emotional valence. Especially, patients with BD having more unstable self-esteem compared with HCs, which couples with unrealistic standards of success as well as emotion dysregulation, has been suggested to make BD patients prone to extremely fluctuating in self-evaluation, finally resulting in their vulnerability to mood instability (38). Another possible area of clinical relevance in this link may be associated with their critical roles in the triple networks (39), combined with our detection of the connectivity between ACC and sPFC in the consensus connection set. Copious amounts of literature have demonstrated the ACC, sMPFC, and sPFC as the key nodes for the salience network (SN), default mode network (DMN), and central executive network (CEN), respectively. In the triple-network framework, it has been proposed that the SN plays a switching role to influence the activity of the CEN and DMN in different mental processing. This study using a machine learning method may extend prior evidence to further document the disrupted switching role of the SN on the DMN and CEN in BD (27).

Limitations of the present study should be pointed out. First, the sample size, totally comprising of 83 patients with BD and 94 HCs, is relatively small from a machine learning perspective and may cause the overfitting problem. Second, our relatively small sample size may degrade the statistical power in the correlation analysis, which showed no significant associations between the behavioral and clinical parameters and the abnormal functional connectivity values in patients. However, consistent with our findings, previous studies (40) also have not found significant correlations between functional connectivity strength and clinical symptoms in BD. Thus, another possible cause is the heterogeneity in mood states and medication exposures of our sample. Nevertheless, future studies using larger samples are needed to clarify the relationship of the abnormal functional connectivity with clinical symptoms in BD. Third, all the patients with BD were treated with medication. The majority of patients took antidepressants, lithium, and/or anticonvulsants, and some patients were also on antipsychotics. Medication administration may influence our findings. Fourth, patients with BD and HCs were not completely matched by age and education years. Although we treated age and education years as covariance factors to exclude their effect, this inconsistency in age and education years distribution between the two groups may confound our results. Fifth, only structural and functional connectivity were recruited, and therefore, more modalities should be used. Future studies with anatomical networks that are constructed using diffusion tensor imaging can be combined with the other two types of brain connectivity in cross-sectional and longitudinal designs and would be beneficial.

## Conclusion

In this study, we employed a semi-multimodal fusion framework by integrating complementary information from structural and resting-state neuroimaging data for distinguishing individual patients with BD from HCs. The convergent findings of the semi-multimodal fusion framework and traditional univariate analyses evidenced the aberrant connectivity between ACC and sMPFC in BD. Further, we allowed more fine granular groups and observed the ACC–sMPFC decoupling exhibited across depression, mania, and remission episodes in BD patients, although the decoupling in mania episodes did not survive after the Tukey–Kramer *post hoc* comparison. Our findings shed light on the decoupling within the anterior midline brain in the pathophysiology of BvD, and this decoupling may serve as a trait marker for this disease.

## Data Availability Statement

The datasets for this study will not be made publicly available due to ethical restrictions, data are available upon reasonable request to Zhening Liu, zningl@163.com.

## Ethics Statement

The studies involving human participants were reviewed and approved by The Second Xiangya Hospital, Central South University. The patients/participants provided their written informed consent to participate in this study. Written informed consent was obtained from the individual(s) and minor(s)’ legal guardian/next of kin, for the publication of any potentially identifiable images or data included in this article.

## Author Contributions

Data Collection: XO, XC, XH. Methodology: JY. Formal analysis: JY. Writing (original draft preparation): JY, WP, XO, ZL. Writing (review and editing): JY, WP, XO, HT, ZL. Project administration: ZL

## Funding

This study was supported by the China Precision Medicine Initiative (2016YFC0906300 to ZL), the National Natural Science Foundation of China (81561168021 to ZL, 81401125 to WP, 81801353 to XO, 81301161 to HT), the Postdoctoral Science Foundation of China (2018M643007 to JY), and the Postdoctoral Research Fund of Xiangya No. 2 Hospital, Central South University (207161 to JY).

## Conflict of Interest

The authors declare that the research was conducted in the absence of any commercial or financial relationships that could be construed as a potential conflict of interest.
