# Supplementary material for: Abnormal Connectivity Within Anterior Cortical Midline Structures in Bipolar Disorder: Evidence From Integrated MRI and Functional MRI
Source: Front Psychiatry. 2019 Oct 29;10:788. doi: 10.3389/fpsyt.2019.00788 (PMC6829675; doi:10.3389/fpsyt.2019.00788)
Supplement: Supplementary file 1 [file Table_1.docx]

**S1. The detailed description of the feature selection procedure.**

We assumed$X=[\begin{matrix} x_{1} & ... & x{}_{i} & ... & x_{n} \end{matrix}]^{T}$ be a $n\times l$matrix that represents *l* features of $n$ training samples, where matrix $X$ is constructed by the individual functional connectivity features extracted from the previous analysis. Let $Y=\left[\begin{matrix} y_{1} & ... & y_{i} & ... & y_{n} \end{matrix}\right]^{T}$be the $n$ dimensional categorical target labels that we aim to predict (+1 = patients with BD; -1 = HCs). The linear regression model used for the prediction can be defined as follows:

$\hat{Y}=XW$ (1)

where $W\in R^{l\times1}$denotes the regression coefficient vector, and $\hat{Y}$indicates the predicted label vector. To estimate the regression coefficient vector, the following objective function should be minimized based on the k-support norm:

$\min_{w} \left\|XW-Y\right\|_{F}^{2}+\lambda_{1}\left(\sum_{i=1}^{k-r-1} \left(\left|W\right|_{i}^{\downarrow}+\frac{1}{r+1}\left(\sum_{i=k-r}^{d} \left|W\right|_{i}^{\downarrow}\right)^{2}\right)\right)^{\frac{1}{2}}$ (2)

where$\lambda_{1}$is the regularization parameter controlling the degree of sparsity in the models, $\left|w\right|_{i}^{\downarrow}$is the *i*-th largest element in the vectors, and *r* is the unique integer in$\left\{\begin{matrix} 0 & \ldots& k-1 \end{matrix}\right\}$satisfying

$\left|w\right|_{k-r-1}^{\downarrow}>\frac{1}{r+1}\sum_{i=k-r}^{d} \left|w\right|_{i}^{\downarrow}\geq\left|w\right|_{k-r}^{\downarrow}$ (3)

As the structural connectivity matrix in our study were obtained on the entire group rather than on a single subject (i.e., the dimensions of the two matrices $\mathbf{a}$nd $M_{S}^{HC}$ are 1×l), we therefore replicated the size of $M_{S}^{BD}$ by the sample size of the patients with BD and the size of $M_{S}^{HC}$ by the sample size of the HCs to construct a new structural connectivity matrix $M_{S}$. Let $M_{S}=\left[\begin{matrix} m_{1} & ... & m_{p} & m_{p+1} & ... & m_{n} \end{matrix}\right]^{T}$be a $n\times l$matrix that denotes the new generated anatomical connectivity, where p is the sample size of the patients with BD. The elements from 1 to *p* row vectors are the same, and the elements from $p+1$ to $n$ row vectors are identical.

In our proposed method, we utilized the complementary information conveyed by the structural connectivity features. We added the following inter-modality distance constraint:

$D=\sum_{i=1}^{n} \left\|x_{i}-m_{i}\right\|_{F}^{2}$ (4)

where $x_{i}$ denotes the feature vectors of the functional connectivity features of the *i*-th subject and $m_{i}$denotes the feature vectors of the anatomical connectivity features of the *i*-th subject. The relative distance between the inter-modality feature vectors is $\left\|x_{i}-m_{i}\right\|_{F}^{2}$. Therefore, the object function of our proposed multi-modality feature selection model can be further defined as follows:

$\min_{w} \left\|XW-Y\right\|_{F}^{2}+\lambda_{1}\left(\sum_{i=1}^{k-r-1} \left(\left|w\right|_{i}^{\downarrow}+\frac{1}{r+1}\left(\sum_{i=k-r}^{d} \left|w\right|_{i}^{\downarrow}\right)^{2}\right)\right)^{\frac{1}{2}}+\lambda_{2}D$ (5)

where $\lambda_{1}>0$ and $\lambda_{2}>0$ control the sparseness and the degree of preservation of the inter-modality relationship, respectively. The proportion of different penalty terms were adjusted by controlling hyperparameter $\lambda_{1}$ and $\lambda_{2}$. For example, we can remove the effects of inter-modality relationship from the “feature selection” step by setting $\lambda_{2}$ =0. By adding $D$, we can obtain a sparser X vector.

**Table S1.** The distribution of consensus connections.

| **Index** | **Region 1** | **Region 2** |
| --- | --- | --- |
| 1 | L ACC | L sMPFC |
| 2 | L ACC | L Superior Frontal Gyrus |
| 3 | L Caudate | L Supp Motor Area |
| 4 | L SupraMarginal | L Postcentral |
| 5 | L Hippocampus | L Middle Cingulum |
| 6 | L Inferior Occipital | R Calcarine |
| 7 | R Amygdala | L Supp Motor Area |
| 8 | L Paracentral Lobule | L Inferior Frontal Oper |
| 9 | L ParaHippocampal | R Middle Cingulum |
| 10 | L Middle Temporal | L ParaHippocampal |
| 11 | R Middle Temporal | L Cuneus |
| 12 | L Putamen | L Supp Motor Area |
| 13 | L Middle Temporal Pole | L Olfactory |
| 14 | R Superior Temporal Pole | L Rectus |
| 15 | L Precuneus | L Angular |
| 16 | L Amygdala | L sMPFC |
| 17 | R Amygdala | L sMPFC |
| 18 | L Caudate | R Inferior Frontal Triangle |
| 19 | R Caudate | R Paracentral Lobule |
| 20 | R Heschl | L Rectus |
| 21 | L Angular | R SupraMarginal |
| 22 | R Angular | L Lingual |
| 23 | R Superior Temporal Gyrus | L Amygdala |
| 24 | R Caudate | L Middle Frontal Orbit |
| 25 | L Angular | L Hippocampus |
| 26 | L Olfactory | R Superior Frontal Gyrus |
| 27 | L Angular | L Calcarine |
| 28 | R ACC | R sMPFC |
| 29 | R Middle Occipital Gyrus | L Lingual |
| 30 | L Middle Temporal Gyrus | R Superior Temporal Gyrus |
| 31 | L Hippocampus | L Supp Motor Area |
| 32 | L Middle Temporal Gyrus | L Medial Frontal Orbit |
| 33 | L Inferior Parietal | L Lingual |
| 34 | R Fusiform | L ParaHippocampal |
| 35 | L Superior Temporal Gyrus | R Heschl |
| 36 | R Caudate | R Precentral |
| 37 | L Middle Temporal Gyrus | R Rectus |
| 38 | L SupraMarginal | R Fusiform |
| 39 | L Lingual Gyrus | L Insula |
| 40 | L Middle Temporal Gyrus | L Precuneus |

†Note: **Consensus connections are arranged in descending order according to their discriminative ability in classification. ACC, anterior cingulate cortex; sMPFC, superior medial prefrontal cortex.**

**Table S2.**  The distribution of consensus nodes.

| **Index** | **Regions** |
| --- | --- |
| 1 | L ACC |
| 2 | L sMPFC |
| 3 | L Supp Motor Area |
| 4 | L Middle Temporal |
| 5 | L Angular |
| 6 | L ParaHippocampal |
| 7 | L Hippocampus |
| 8 | L Caudate |
| 9 | R Amygdala |

†Note: **Consensus nodes are arranged in descending order according to their discriminative ability in classification. ACC, anterior cingulate cortex; sMPFC, superior medial prefrontal cortex.**

**Table S3.** After controlling for age, gender, and educational years, functional connections showing significantly altered connections strength in patients with BD type I compared to HC.

| **Connections** | **Patients with BD type I**  **(N =79)** | **Healthy Controls**  **(N = 94)** | ***P* value** | **FDR corrected** |
| --- | --- | --- | --- | --- |
| **Corrected Structural Connections** | | | | |
| None |  |  |  |  |
| **Corrected Functional Connections** | | | | |
| L ACC--- L sMPFC | -0.1 ±0.23 | 0.09±0.23 | <0.0001 | 0.0017 |
| R ACC---L sMPFC | -0.09±0.27 | 0.08±0.26 | <0.0001 | 0.0368 |
| L ACC--- R sMPFC | -0.09±0.25 | 0.7±0.24 | <0.0001 | 0.0365 |
| R ACC---R sMPFC | -0.1±0.22 | 0.08+0.29 | <0.0001 | 0.0492 |

†Note: FDR corrected using Benjamini and Hochberg method.

‡Abbreviation: L, left; R, right; ACC, anterior cingulate cortex; sMPFC, superior medial prefrontal cortex.


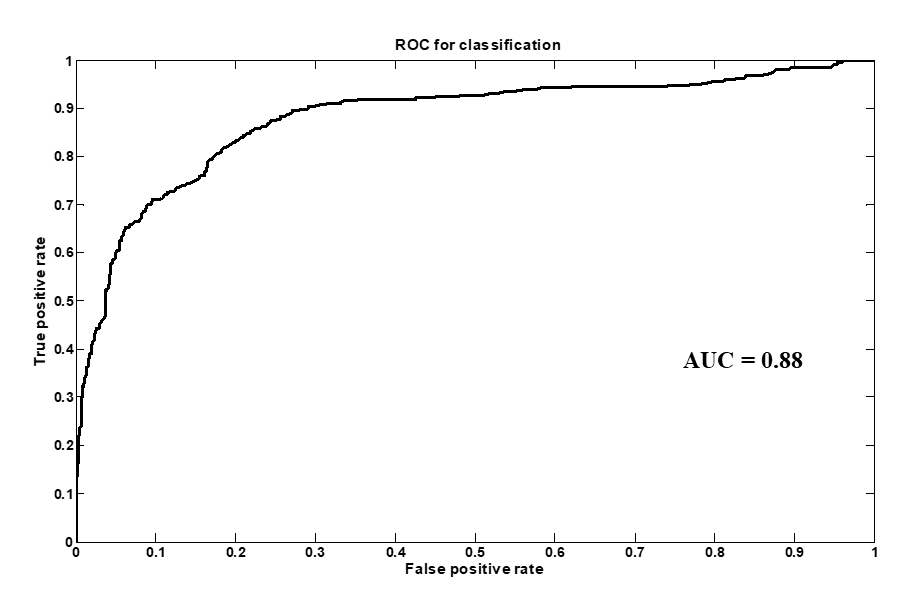


**Figure S1.** ROC curves of the classification.
